# Implant stability in the posterior maxilla: clinical and radiographic comparison of osseodensification and conventional drilling: a randomized clinical trial

**DOI:** 10.1007/s00784-025-06526-8

**Published:** 2025-09-29

**Authors:** Sara Amr Abdelraouf, Omnia Aboul Dahab, Basma Mostafa, Sarah Mohammed Kenawy, Omnia K. Tawfik

**Affiliations:** 1 Oral Medicine and Periodontology Department, Faculty of Dentistry, Cairo University, Cairo, Egypt; 2 Oral and Maxillofacial Radiology Department, Faculty of Dentistry, Cairo University, Cairo, Egypt; 3 Surgery and Oral Medicine Department, Oral and Dental Research Institute, National Research Centre, Cairo, Egypt

**Keywords:** Osseodensification, Densah burs, Conventional, Standard drills, Implant stability, Crestal bone

## Abstract

**Objectives:**

The aim of this randomized clinical trial was to clinically and radiographically compare the effect of osseodensification (OD) and conventional drilling (CD) on implant stability in the posterior maxilla.

**Materials and methods:**

Twenty patients received 20 implants after being randomly assigned for osteotomy preparation with either OD (test) (*n* = 10) or CD (control) (*n* = 10). Implant stability quotient (ISQ) and crestal bone loss were monitored closely from implant insertion through 12 months of loading. Insertion torque and implant survival were also assessed during the study.

**Results:**

In OD group, one patient was lost to follow up and all other implants were in Function after 12 months of loading (9/9), while only 8/10 implants survived in CD group. OD was associated with significantly higher mean ISQ values; post-insertion and during the 1st month of healing, compared to CD. A high relatively unchanged stability was observed throughout osseointegration with OD method, while a stability dip occurred during the 2nd and 3rd weeks of healing in CD group. There was no significant difference in crestal bone loss and insertion torque between groups.

**Conclusions:**

Within the limitations of this study, OD seems to provide earlier implant stability in terms of ISQ values, and may improve survival rates in the posterior maxilla, compared to CD, with no negative impact on crestal bone after 12 months of implant loading. ClinicalTrials.gov Identifier: *NCT04442763* (registration date 15/6/2020).

**Clinical relevance:**

OD may be used as an alternative to CD to achieve earlier implant stability in the posterior maxilla.

**Supplementary Information:**

The online version contains supplementary material available at 10.1007/s00784-025-06526-8.

## Introduction

Placing implants in the posterior maxilla poses a challenge due to the inherent poor bone quality. Therefore, implant stability is critical for success in this anatomical region, encompassing primary stability, a product of mechanical implant-to-bone engagement at insertion, and secondary stability, a biological process driven by progressive bone remodeling around the implant [1].

Achieving optimal primary stability and maintaining it during the initial healing stages is a key for predictable osseointegration and early loading. This is influenced by factors like bone quality, implant geometry, and most importantly, the surgical technique that minimizes bone trauma during osteotomy [2, 3]. Various osteotomy techniques have been proposed to improve the relationship between the implant and the instrumented bone walls, influencing remodeling and the rate of secondary stability [4].

The transition from primary to secondary stability is not linear, with a stability dip often occurring during early healing due to bone remodeling to surgical trauma [5–8]. Consequently, if stability at implantation is high, a minor decrease during initial healing typically does not affect the clinical outcome. However, in case of low primary stability, any reduction in stability could potentially compromise implant success or delay loading, which impacts treatment time and cost [8].

Osseodensification (OD); a proposed bone-preserving osteotomy technique, has shown promise over conventional drilling (CD) by enhancing implant stability in areas of poor bone density [9]. Unlike traditional drilling, OD condenses bone into the implant osteotomy walls using specially designed “Densah burs” rotating counterclockwise. It has been suggested that with OD, densifying peri-implant bone is achieved through controlled condensation and elastic deformation that allows bone to release strains by springing back around the inserted implant. This enhances bone-to-implant contact and provides high primary stability with minimal bone trauma, potentially improving bone remodeling during early healing. Furthermore, bone neo-formation might be accelerated by the presence of auto-grafted bone matrix, cells, and biochemicals along the osteotomy, facilitating a seamless transition to osseointegration [10–15].

Given the limited evidence on OD use in low-density bone, related primarily to pre-clinical studies and a few randomized clinical trials (RCTs), the impact of OD on implant stability during healing needed further investigation. Therefore, the primary aim of this RCT was to compare the effect of OD and CD on implant stability changes, assessed through implant stability quotient (ISQ); during osseointegration and after 12 months of loading in the posterior maxilla. Secondary outcomes included insertion torque, crestal bone loss and implant survival.

## Materials and methods

The current study was designed as a parallel-group randomized clinical trial with a 1:1 allocation ratio and was executed at the Periodontology Department, Faculty of Dentistry, Cairo University. The protocol was approved by the Ethics Committee of Scientific Research (February 2020/ approval number: 11.2.20), registered in ClinicalTrials.gov (NCT04442763), and followed the CONSORT guidelines [16] (Online Resource 1).

The Sample size calculation, which was based on expert opinion and a previous study [17], used ISQ as the primary outcome. With a mean and SD of 56 ± 2 for the control group, a minimal clinically significant difference of 3, an 80% power, and a 5% significance level, 16 implants were needed. The sample was increased to 20 implants to account for a 25% dropout rate, calculated using PS software^1^.

This study included 20 patients with single bounded implant sites in the maxillary premolar-molar area. Subjects were recruited by S.A.A. from the Periodontology Clinic, Faculty of Dentistry, Cairo University between January 2021 and August 2022 and were monitored for 12 months after loading.

Eligible participants were at least 18 years old, medically healthy, cooperative, and with good oral hygiene. They had a pristine healed alveolar ridge with at least a 6-month post-extraction period, with sufficient dimensions (width ≥ 6 mm, height ≥ 10 mm) and opposing dentition. Exclusion criteria included active periodontal disease, para-functional habits, head/neck radiation history, smoking, and pregnancy or lactation. 

Clinical and radiographic examinations were performed, including a pre-operative assessment of bone density through visual evaluation of bone morphology on CBCT images, to include only sites with D3 and D4 bone quality according to Misch’s classification [18]. Enrolled participants signed an informed consent after being fully informed about the study procedures and follow-up and they received supra-gingival scaling and plaque control instructions one week before surgery.

Participants were randomly assigned by B.M. to the OD (test) group or CD (control) group using simple computer-generated randomization^2^ with a 1:1 allocation ratio. Allocation was concealed in identical, opaque sealed envelopes and revealed by O.K.T on the surgery day. The study was double-blinded, including participants, outcome assessors and the statistician. Blinding the principal investigator was not feasible due to the different drilling systems used.

**Fig. 1:**
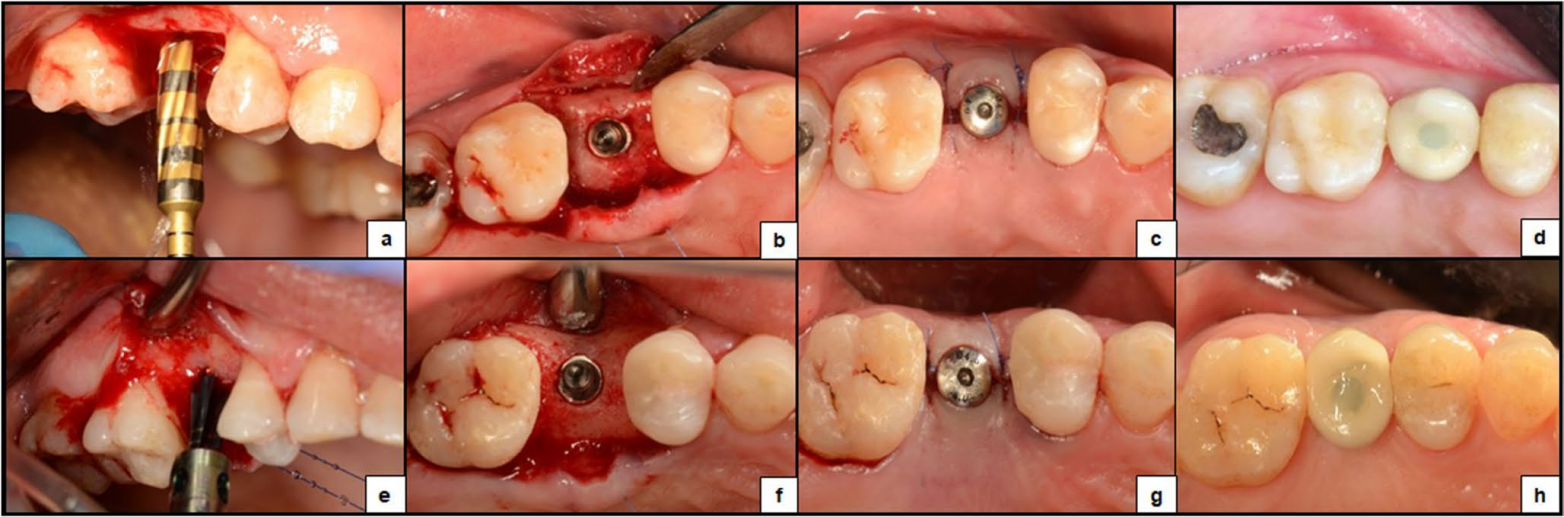
Surgical and prosthetic steps – Osseodensification case (**a**: osteotomy preparation with Densah burs, **b**: implant placement, **c**: suturing around healing abutment and **d**: final prosthesis) – Conventional drilling case (**e**:osteotomy preparation using standard drills, **f**: implant placement, **g**: suturing around healing abutment and **h**: final prosthesis)

The surgical procedures were performed by S.A.A. In both groups, after raising a minimally-invasive full-thickness flap under infiltration anesthesia^3^, osteotomy preparation was carried out at 800–1200 rpm, with a pumping action and saline irrigation and drilling was initiated using a pilot drill, rotated in a clockwise direction. Afterwards, in the test group, OD drilling was performed sequentially using Densah burs^4^ in an anti-clockwise direction, following the densifying reference guide (Fig. 1-a). While in the control group, standard drilling was done with clockwise rotation, following the manufacturer guidelines (Fig. 1-e). Implants were inserted, first using a motor hand-piece at 25–35 rpm, and then with a manual torque wrench^5^ (Fig. 1-b&f), with which, insertion torque (IT) was measured in Newton Centimeter (N.cm). Healing abutments were attached to allow access to implants during follow ups, and the flap was approximated with interrupted sutures (Fig. 1-c&g). 

Participants received antibiotics (amoxicillin 1 g) and anti-inflammatory drugs (Ibuprofen 400 mg) twice daily for 5 days. For penicillin allergic patients, the alternative protocol was clindamycin 600 mg every 8 hours for 3 days. Patients were advised to follow self-care instructions and to revisit for suture removal after 1 week. Three months after implant placement, and ensuring that each implant reached an ISQ value of ≥ 70, final screw-retained crowns were fabricated and implants were loaded [19] (Fig. 1-d&h).

The implant system^6^ used in this study featured an osteo-conductive SLA-coated surface and a tapered body with buttress threads. It included a crestal macro-thread design with a 0.8 mm pitch and a self-compactable, dome-shaped apex. Additionally, the implant incorporated a platform-switching design and a conical/hex internal connection. Implants size was restricted to diameters of 3.5–4 mm and lengths of 10–11.5 mm.

Implant stability (primary outcome) was assessed in terms of ISQ values using a smart peg^7^ and an Osstell device^8^ at multiple time points; post-insertion, after 1,2,3,4,6,8 weeks, 3 months and after 12 months of prosthetic loading. ISQ values were recorded as the average of buccal and mesial readings taken twice [19].

**Fig. 2:**
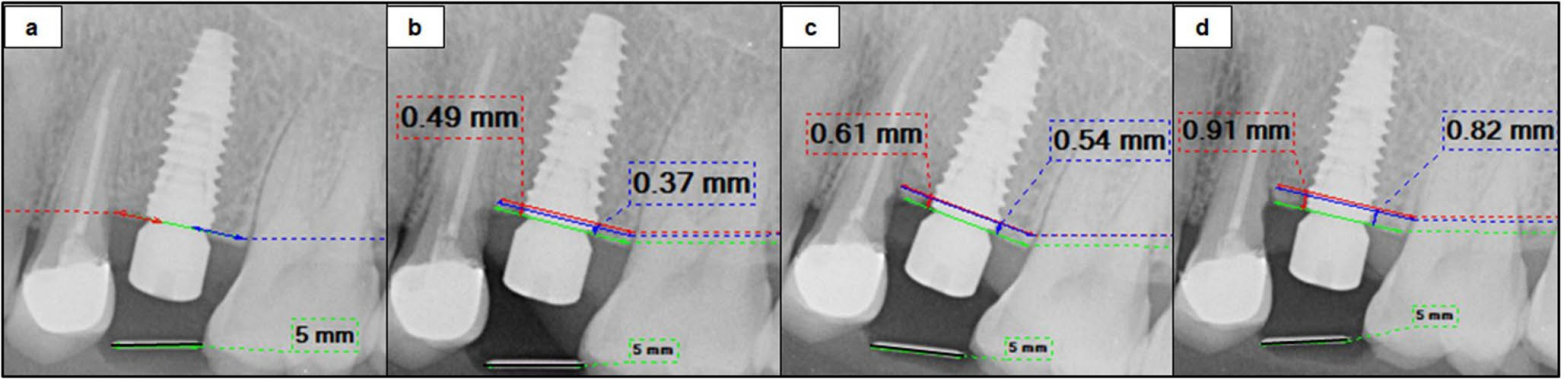
Radiographic assessment of crestal bone loss from implant platform (green line) to the first visible bone-to-implant contact; mesially (red line) and distally (blue line), at each time point (**a**: post-insertion, **b**: 3 months, **c**: 6 months post-loading and **d**: 12 months post-loading)

Standardized digital periapical radiographs were taken four times using the paralleling technique and a customized stent: post-insertion, at 3 months, and after 6 and 12 months of prosthetic loading (crowns were removed and healing abutments were placed for adequate seating of the stent). Radiographs were analyzed with DIGORA software^9^, to measure crestal bone loss in millimeters from the implant platform to the first bone-to-implant contact (Fig. 2). The final value was the average of mesial and distal measurements [19]. Implant survival was recorded up to 12 months of loading according to the criteria established by ICOI Pisa Consensus Conference [20] as follows: (a) no pain or tenderness during function (b) absence of mobility (c) radiographic crestal bone loss of 2–4 mm (d) no history of exudation.

### Statistical analysis

Data were presented as mean, standard deviation, median, range, frequencies, and percentages. Normality was assessed using Kolmogorov-Smirnov and Shapiro-Wilk tests. For parametric data, repeated measures ANOVA compared inter and intra-group data of ISQ, followed by Bonferroni’s post-hoc test when significant, while IT was analyzed using Student’s t-test. For non-parametric data as crestal bone loss, Mann-Whitney U test was used for inter-group comparisons, and Friedman’s and Dunn’s tests for intra-group comparisons. Fisher’s Exact test analyzed implant survival. Significance was set at *P* ≤ 0.05, and analysis was performed with statistical software^10^.

## Results

A flowchart describing the study phases is shown in Online Resource 2. Out of the 20 enrolled participants, 3 were excluded (1 dropout in the OD group and 2 early implant failures in the CD group). Thus, 17 participants (4 males, 13 females; mean age 45.2 ± 7.4 years) completed 12 months of prosthetic loading and were included in the final analysis. Demographic and implant-related data are presented in Online Resource 3, which shows that there was no statistically significant difference in gender distribution, implant size or location between groups.

Implant stability quotient results are presented in Table 1-A. The OD group had a mean ISQ of 72.7 ± 3.1 post-insertion. From 1 week to 3 months (week 1: 69.4 ± 2.6, 3 months: 75.8 ± 1.9), there was no significant change in ISQ values compared to post-insertion, followed by a significant increase to 77.7 ± 1.5 after 12 months of loading. While in the CD sites, post-insertion stability had a mean ISQ of 63.3 ± 12, with no significant change in the 1st post-insertion week (62.6 ± 9.2). Mean ISQ values significantly declined during the 2nd (60.3 ± 6.3) and 3rd (59.9 ± 4.6) weeks, then they significantly increased from the 4th week (62.3 ± 6.6) to 12 months of loading (75.8 ± 3.5). Inter-group comparisons showed that OD had significantly higher mean ISQ values than CD; immediately post-insertion and after 1, 2, 3, and 4 weeks. However, after 6, 8 weeks, 3 months, and 12 months of loading, the difference between groups was not statistically significant.

**Table 1:** Implant stability quotient changes and insertion torque in both groups

(A) Implant stability quotient (ISQ)		Time	OD (*n* = 9)	CD (*n* = 8)		
			Mean ± SD	Mean ± SD	*P* -value	*Effect size* (*Partial Eta squared*)
		Post-insertion	72.7^bc^ ± 3.1	63.3^e^ ± 12	0.037*	0.258
		week-1	69.4^c^ ± 2.6	62.6^e^ ± 9.2	0.049*	0.234
		week-2	68.6^c^ ± 3.1	60.3^f^ ± 6.3	0.003*	0.448
		week-3	68.2^c^ ± 4.7	59.9^f^ ± 4.6	0.002*	0.476
		week-4	68.7^c^ ± 4.7	62.3^e^ ± 6.6	0.036*	0.262
		week-6	72.2^c^ ± 3.2	67.5^d^ ± 8.5	0.145	0.136
		week-8	74.1^b^ ± 1.9	69.4^c^ ± 8.4	0.119	0.154
		3 months	75.8^b^ ± 1.9	73.8^b^ ± 4.7	0.237	0.092
		12 months PL	77.7^a^ ± 1.5	75.8^a^ ± 3.5	0.155	0.130
		*P* -value	0.001*	0.001*		
		*Effect size* (*Partial Eta squared*)	0.918	0.930		
(B) Insertion torque (N.cm)	Mean ± SD N.cm ≥ 35[n, (%)]		OD (*n* = 9)	CD (*n* = 8)	*P* -value	
			38.3 ± 7.1 8 (88.9%)	30.6 ± 9.4 4 (50%)	0.074	

*OD* osseodensification, *CD* conventional drilling, *PL* post-loading, *N.cm* Newton centimeter

*:Significant at *P *≤0.05, Different superscripts in a column indicate statistically significant change by time

Mean IT was 38.3 ± 7.1 N.cm in OD group and 30.6 ± 9.4 N.cm in CD group, with no significant difference (*P*-value = 0.074). Using 35 N.cm as the cut-off, 8 out of 9 implants (88.9%) in OD group and 4 out of 8 implants (50%) in CD group had IT of ≥ 35 N.cm (Table 1-B).

Both groups showed a statistically significant increase in median crestal bone loss from implant insertion (OD: 0 (0,0.17), CD: 0 (0,0.21)) to 12 months of loading (OD: 1 (0.85,1.51), CD: 1.018 (0.8,2.4)) (*P*-value < 0.001). Inter-group comparisons of bone loss showed no significant difference between groups at any time point during the study (Table 2-A).

**Table 2:** Crestal bone loss and implant survival in both groups

(A) Crestal bone loss (mm)		OD (*n* = 9)	CD (*n* = 8)			
	Time	Median (Range)	Median (Range)	*P*-value	*Effect size (d)*	
	Post-insertion	0 (0,0.17)^c^	0 (0,0.21)^d^	0.864	0.047	
	3 months	0.72 (0.45,1.13)^b^	0.6 (0.41,1.8)^c^	0.441	0.38	
	6 months PL	0.77 (0.65,1.4)^b^	0.853 (0.57,2.08)^b^	0.665	0.211	
	12 months PL	1 (0.85,1.51)^a^	1.018 (0.8,2.4)^a^	0.962	0.023	
	*P* -value	< 0.001*	< 0.001*			
	*Effect size (w)*	1	1			
(B) Implant survival [n, (%)]		OD (*n* = 9)	CD (*n* = 10)	*P*-value		*Effect size* (Odds Ratio)
	Survival	9 (100%)	8 (80%)	0.474		2.125
	Failure	0 (0%)	2 (20%)			

*OD* osseodensification, *CD* conventional drilling, *PL* post-loading

*:Significant at *P* ≤ 0.05, Different superscripts in a column indicate statistically significant change by time

After 12 months of loading, all implants in the OD group (9/9) were successfully osseointegrated, with a 100% survival rate. In the CD group, the survival rate was 80%, since 2 out of 10 implants failed to osseointegrate, with no significant difference between groups (*P* = 0.474). The odds of implant survival were 2.125 times higher in the OD group (Odd’s ratio: 2.125) (Table 2-B). All implants in this study demonstrated crestal bone loss of < 2 mm, except for one implant in the CD group, which showed a loss of 2.4 mm. Apart from this, no adverse events or complications were observed in either group throughout the study.

## Discussion

Osteotomy preparation protocols influence the kinetics of peri-implant bone healing. They are crucial for implant stability and osseointegration, particularly in the posterior maxilla, where bone quality is typically suboptimal [4]. In the present study, the predominance of female participants may have further compromised bone quality [21]. However, their uniform representation among groups ensured homogeneity, with the sample falling within a similar bone density range, thereby minimizing the potential influence of gender distribution on the study outcomes. Moreover, to solely test the effect of osteotomy preparation, implants with a pre-determined size range were utilized, where available ridge dimensions permitted their even allocation across groups, while avoiding bi-cortical anchorage, which limited the impact of these factors on implant stability.

Only a handful of studies [22, 23] have monitored implant stability over time in conjunction with OD, therefore, implant stability maturation was chosen as the primary outcome of this study as it reflects the osseointegration process around implants. CD sites exhibited lower primary ISQ values compared to OD, with a significant stability dip taking place during the second and third weeks of healing, consistent with the literature linking this dip to the peri-implant bone resorption induced by surgical trauma, leading to a rapid loss of mechanical stability that is not compensated by the developing biological stability [5–8]. While in the OD group, the high ISQ values recorded at insertion did not significantly change throughout the course of osseointegration.

The different stability patterns observed during early healing help explain the superiority of OD over CD in enhancing ISQ values at implant insertion and during the first month of healing. Later, however, both groups showed comparable mean ISQ values through the follow up period, indicating that following the stability dip, CD stability increased over time, eventually catching up with OD. This recovery was reinforced by the predominance of new bone apposition onto the implant surface and the development of secondary stability [5–8]. In addition, a significant stability increase was observed in both groups after prosthetic loading, which can be related to bone straining, adaptation and maturation in response to functional loads [24].

It seems that OD may offer advantages over CD, not only by bone bulk preservation which contributes to high mechanical stability [22, 23, 25–31], furthermore, by minimizing peri-implant bone trauma or accelerating bone neo-formation, resulting in a limited stability decrease during early healing. Previous preclinical studies suggest that the cancellous bone condensation and compaction-auto-grafting provided by OD optimize peri-implant bone density by applying controlled compressive stress within the bone’s elastic limits. This creates a viscoelastic stress relaxation of bone against the inserted implant, releasing residual strains and maximizing bone-to-implant contact. Accordingly, implant physical interlocking may be improved without the excessive bone straining that can lead to significant remodeling and stability loss. Furthermore, histological analyses have shown pre-osteoblasts and bio-chemicals in the auto-grafted bone particles, acting as nucleation sites for new bone formation, which may accelerate the transition to biological stability and speed up osseointegration [10–15, 32]. The above-stated mechanism may also justify the higher survival rate of the OD-inserted implants, which had double the likelihood of survival than CD-inserted ones, with two early failures recorded.

Similar to the present findings, Stacchi et al. [23] did not observe a meaningful decrease in ISQ values in their OD group over 90 days of healing. However, Bergamo et al. [22] reported a significant stability drop at 3 weeks in the OD sites. The higher IT and ISQ values observed in their study, as well as the different pattern of bone remodeling, may be attributed to the use of implants with varying designs and larger dimensions. Additionally, their OD group showed superior stability over CD at insertion, 3 weeks, as well as 6 weeks. The 6-week disagreement may arise from under-sizing their CD osteotomies, leading to excessive remodeling and delayed secondary stability [33].

An insertion torque of 35 N.cm and/or an ISQ of 65 is generally considered sufficient for immediate loading, ensuring primary implant stability to withstand forces without the risk of micro-motion that can disrupt healing and osseointegration [34–36]. However, raising ISQ values to 70 is recommended for better long-term outcomes and reduced implant failure risk, particularly in low bone quality or high-demand patients [37–39]. In the current investigation, the majority of the OD group achieved an IT of ≥ 35 N.cm, in contrast to only half of the CD group. As for ISQ, implants installed using OD had a primary stability that exceeded the aforementioned thresholds (mean ISQ: 72.7 ± 3.1), and even during the lowest stability period (mean ISQ at week-3: 68.2 ± 4.7), ISQ values remained on the higher end of the above-stated range, suggesting that OD may be a suitable technique for immediate loading to shorten the overall treatment period. Meanwhile, implants in the CD-prepared sites achieved the recommended value for load-bearing capacity probably after 8 weeks of insertion (mean ISQ at week-8: 69.4 ± 8.4).

Consistent with a recent meta-analysis [9], crestal bone loss was observed in both groups, likely due to factors such as surgical trauma, biological width development, micro-gaps at the implant-abutment interface, and loading-related biomechanics [40]. Repeated access to implants during follow-ups may have also contributed [41]. However, bone loss was similar in the two groups throughout the study, negating a negative impact of OD on crestal bone. After 12 months of loading, the amount of bone loss in both groups fell within the range considered successful by the literature [20, 42].

This study had some limitations, including a small sample size, eligibility restricted to medically healthy patients, and the use of a single implant type with a pre-decided size limit. Besides, the study did not include a quantitative assessment of bone density, which could have helped evaluate the influence of bone density on implant stability patterns and provided a standardized measure of the OD’s effect on the surrounding bone quality. These factors should be considered before generalizing the findings of this study.

## Conclusion

Within the study’s limitations, OD seems to offer earlier implant stability; as indicated by ISQ values, and may improve survival rates in the posterior maxilla compared to CD, with no adverse effects on crestal bone after 12 months of loading. However, both osteotomy techniques resulted in similar implant insertion torques. Further clinical trials with larger sample size, quantitative bone density evaluations, various implant designs and sizes, and different loading protocols are needed to fully assess OD’s potential as an alternative in routine clinical practice.

## Supplementary Information

Below is the link to the electronic supplementary material.

Supplementary Material 1

Supplementary Material 2

Supplementary Material 3

## Data Availability

Data supporting findings of this study are available from corresponding author upon reasonable request.

## Footnotes

1 Power and Sample Size Calculation Software Version 3.1.2 (Vanderbilt University, Nashville, Tennessee, USA).

2 www.random.org, Randomness and Integrity Services Ltd.

3 Artinibsa 40 mg/0.01 mg/ml local anesthetic agent, Inibsa, S.A, Spain.

4 Densah Burs, Versah, Jackson, MI, USA.

5 Torque Wrench, Neobiotech Co., Seoul, Korea.

6 Neobiotech IS-II Active, Neobiotech Co., Seoul, Korea.

7 SmartPeg (TM), Integration Diagnostics Ltd., Goteborg, Sweden.

8 Mega ISQ, Osstell AB, Integration Diagnostic AB, Stampgatan, Goteborg, Sweden.

9 Digora for Windows 2.5™, SOREDEX Inc., Tuusula, Finland.

10 IBM SPSS Statistics for Windows, Version 23.0, Armonk, NY: IBM Corp.

## Declarations

### Clinical trial registration

The study was registered in ClinicalTrials.gov (NCT04442763).
