# Supplementary material for: Implant stability in the posterior maxilla: clinical and radiographic comparison of osseodensification and conventional drilling: a randomized clinical trial
Source: Clin Oral Investig. 2025 Sep 29;29(10):480. doi: 10.1007/s00784-025-06526-8 (PMC12477082; doi:10.1007/s00784-025-06526-8)
Supplement: Supplementary file 2 — Supplementary Material 2 [file 784_2025_6526_MOESM2_ESM.docx]

IMPLANT STABILITY IN THE POSTERIOR MAXILLA: CLINICAL AND RADIOGRAPHIC COMPARISON OF OSSEODENSIFICATION AND CONVENTIONAL DRILLING: A RANDOMIZED CLINICAL TRIAL

CLINICAL ORAL INVESTIGATIONS

**Authors**: Sara Amr Abdelraouf^1,3^ MSc,

Omnia Aboul Dahab^1^ PhD,

Basma Mostafa^3^ PhD,

Sarah Mohammed Kenawy^2^ PhD,

Omnia K Tawfik^1^ PhD

**Author affiliation**:

**^1^**Oral Medicine and Periodontology Department, Faculty of Dentistry, Cairo University, Cairo, Egypt

**^2^**Oral and Maxillofacial Radiology Department, Faculty of Dentistry, Cairo University, Cairo, Egypt

**^3^**Surgery and Oral Medicine Department, Oral and Dental Research Institute, National Research Centre, Cairo, Egypt

**Corresponding author:**

Sara Amr Abdelraouf , PhD Candidate, Oral Medicine and Periodontology Department, Faculty of Dentistry, Cairo University, 11 El-Saraya St., Manial, Cairo, Egypt.

sara.amr@dentistry.cu.edu.eg

**
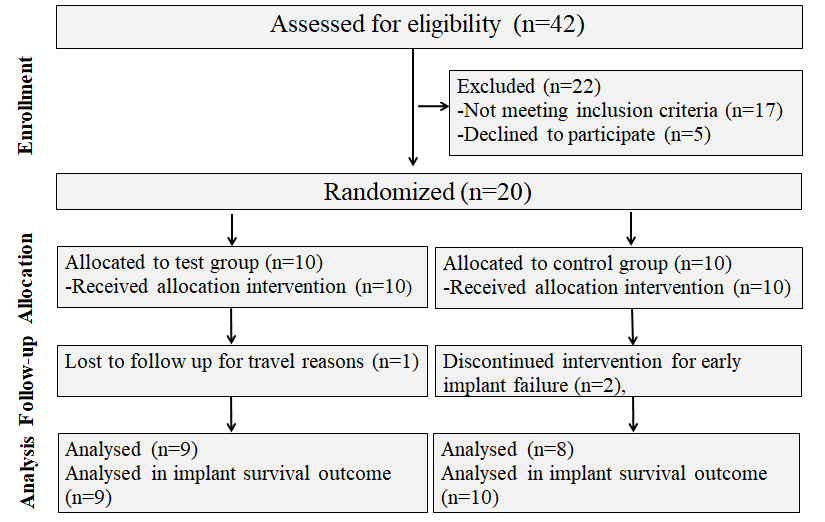
**

**ONLINE RESOURCE 2:** CONSORT diagram of the progress through the phases of the study (enrollment, allocation, follow-up and analysis)
