# Supplementary material for: Implant stability in the posterior maxilla: clinical and radiographic comparison of osseodensification and conventional drilling: a randomized clinical trial
Source: Clin Oral Investig. 2025 Sep 29;29(10):480. doi: 10.1007/s00784-025-06526-8 (PMC12477082; doi:10.1007/s00784-025-06526-8)
Supplement: Supplementary file 3 — Supplementary Material 3 [file 784_2025_6526_MOESM3_ESM.docx]

IMPLANT STABILITY IN THE POSTERIOR MAXILLA: CLINICAL AND RADIOGRAPHIC COMPARISON OF OSSEODENSIFICATION AND CONVENTIONAL DRILLING: A RANDOMIZED CLINICAL TRIAL

CLINICAL ORAL INVESTIGATIONS

**Authors**: Sara Amr Abdelraouf^1,3^ MSc,

Omnia Aboul Dahab^1^ PhD,

Basma Mostafa^3^ PhD,

Sarah Mohammed Kenawy^2^ PhD,

Omnia K Tawfik^1^ PhD

**Author affiliation**:

**^1^**Oral Medicine and Periodontology Department, Faculty of Dentistry, Cairo University, Cairo, Egypt

**^2^**Oral and Maxillofacial Radiology Department, Faculty of Dentistry, Cairo University, Cairo, Egypt

**^3^**Surgery and Oral Medicine Department, Oral and Dental Research Institute, National Research Centre, Cairo, Egypt

**Corresponding author:**

Sara Amr Abdelraouf , PhD Candidate, Oral Medicine and Periodontology Department, Faculty of Dentistry, Cairo University, 11 El-Saraya St., Manial, Cairo, Egypt.

sara.amr@dentistry.cu.edu.eg

**ONLINE RESOURCE 3: Demographic and implant-related data in the two groups**

|  | | | **OD(n=9)** | | **CD(n=8)** | **P-value** |
| --- | --- | --- | --- | --- | --- | --- |
| **Gender**  **[n,(%)]** | | **Male**  **Female** | **2(22.22%)**  **7(77.78%)** | | **2(25%)**  **6(75%)** | **1** |
| **Age**  **Mean±SD** | |  | **45.2 ±7.4 years** | | |  |
| **Implant size**  **[n,(%)]** | **3.5×10**  **4×10**  **4×11.5** | | | **2(22.2%)**  **6(66.7%)**  **1(11.1%)** | **2(25%)**  **5(62.5%)**  **1(12.5%)** | **1** |
| **Implant location [n,(%)]** | | **First premolar**  **Second premolar**  **First molar** | | **2(22.2%)**  **4(44.4%)**  **3(33.3%)** | **3(37.5%)**  **3(37.5%)**  **2(25%)** | **1** |

*Abbreviations: OD: osseodensification, CD: conventional drilling*
